# *FBXW7* Mutations in Patients with Advanced Cancers: Clinical and Molecular Characteristics and Outcomes with mTOR Inhibitors

**DOI:** 10.1371/journal.pone.0089388

**Published:** 2014-02-19

**Authors:** Denis L. Jardim, Jennifer J. Wheler, Kenneth Hess, Apostolia M. Tsimberidou, Ralph Zinner, Filip Janku, Vivek Subbiah, Aung Naing, Sarina A. Piha-Paul, Shannon N. Westin, Sinchita Roy-Chowdhuri, Funda Meric-Bernstam, David S. Hong

**Affiliations:** 1 Department of Investigational Cancer Therapeutics (Phase I Clinical Trials Program), The University of Texas MD Anderson Cancer Center, Houston, Texas, United States of America; 2 Department of Biostatistics, The University of Texas MD Anderson Cancer Center, Houston, Texas, United States of America; 3 Department of Pathology, The University of Texas MD Anderson Cancer Center, Houston, Texas, United States of America; 4 Department of Gynecologic Oncology and Reproductive Medicine, The University of Texas MD Anderson Cancer Center, Houston, Texas, United States of America; University of California Davis, United States of America

## Abstract

**Purpose:**

*FBXW7* is a tumor suppressor gene responsible for the degradation of several proto-oncogenes. Preclinical data suggest that *FBXW7* mutations sensitize cells to mTOR inhibitors. Clinicopathologic characteristics of cancer patients with *FBXW7* mutations and their responses to mTOR inhibitors remain unknown.

**Methods:**

Using multiplex gene panels we evaluated how the *FBXW7* mutation affected the cancer phenotype of patients referred to a phase I clinic starting January 2012. Whenever possible patients positive for FBXW7 mutation were treated with regimens containing an mTOR inhibitors and their outcomes were reviewed.

**Results:**

*FBXW7* mutations were detected in 17 of 418 patients (4.0%). Among tumor types with more than 10 patients tested, *FBXW7* mutations occurred in colorectal cancer (7/49; 14.3%), squamous cell cancer of head and neck (2/18; 11.1%), liver (1/13; 7.7%), and ovarian cancers (1/40; 2.5%). No one clinical, pathological or demographic feature was characteristic of the *FBXW7*-mutated patient population. The mutation occurred in isolation in only 2/17 (12%) patients, and *KRAS* was frequently found as a concomitant mutation, especially in patients with colorectal cancer (6/7; 86%). Ten patients were treated on a protocol containing an mTOR inhibitor, with a median time to treatment failure of 2.8 months (range, 1.3–6.8). One patient with liver cancer (fibrolamellar subtype) continues to have a prolonged stable disease for 6.8+ months.

**Conclusion:**

In patients with advanced cancers, somatic mutations in *FBXW7* usually occur with other simultaneous molecular aberrations, which can contribute to limited therapeutic efficacy of mTOR inhibitors.

## Introduction

The identification of molecular aberrations that are predictive of response to targeted therapy has been the focus of intensive research. Preclinical data from numerous cancer cell lines and mice models have correlated specific genetic mutations with susceptibility to agents inhibiting the pathway putatively activated in the mutated state. [1], [2]. Indeed, major therapeutic advances have recently been made in oncology tailoring treatment to molecular characteristics of some tumors.[3]–[7] Additionally, the strategy of matching druggable genetic abnormalities with targeted agents has demonstrated efficacy in umbrella protocols. [8], [9] However, much remains unknown regarding the efficacy of novel targeted agents and how genetic alterations can be translated to the clinic, and current preclinical models are incomplete. [10].

Extensive comprehensive molecular profiling is commercially available for cancer patients and some results suggest potential treatment options based exclusively on the mutations found in tested tumors. Establishing a correlation between the preclinical activity of targeted agents with clinical data is essential to optimize this approach.

*FBXW7* is a tumor suppressor gene that is mutated in various human tumors. [11] This gene encodes a F-box protein responsible for ubiquitination and turnover of several oncoproteins and its loss of function has been associated with genetic instability and tumor growth. [12], [13] mTOR is one of the substrates of *FBXW7*-mediated protein degradation, and loss of function of *FBXW7* increases the levels of total and activated mTOR. [14] Preclinical data have suggested that inactivating mutations of *FBXW7* could predict sensitivity to the mTOR inhibitor rapamycin,. [14], [15]; however, their clinical utility remains unknown.

Therefore, we investigated the *FBXW7* mutational status and clinical and demographic characteristics of patients with advanced cancer referred to our Phase I Clinical Trials Program and the outcomes of such patients treated with agents targeting the mTOR pathway.

## Patients and Methods

### Patients

We reviewed the electronic medical records of all patients with advanced solid tumors tested for *FBXW7* mutations referred to the Department of Investigational Cancer Therapeutics (Phase I Clinical Trials Program) at The University of Texas MD Anderson Cancer Center starting in January 2012. Patients who tested positive for *FBXW7* mutations were included in further analyses. Patients with colorectal cancer who tested negative for *FBXW7* mutations were included as controls for the colorectal cancer subgroup. This study and all associated treatments were conducted in accordance with the guidelines of the MD Anderson Institutional Review Board (IRB). This study was part of an umbrella protocol approved by MD Anderson IRB. The need for written informed consent was waived due to the retrospective nature of the study.

### Tissue Samples and Mutation Analysis

*FBXW7* mutations were investigated in archival formalin-fixed, paraffin-embedded tissue blocks or material from fine needle aspiration biopsies obtained from diagnostic and/or therapeutic procedures. All histologies were centrally reviewed at MD Anderson. *FBXW7* mutation analysis was performed in different Clinical Laboratory Improvement Amendment-certified laboratories as part of a gene panel analysis. These included 182 genes in targeted next-generation sequencing Foundation One platform (Foundation Medicine, Cambridge, MA), 46 genes in Ion Torrent next-generation sequencing (Baylor’s Cancer Genetics Laboratory, Houston, TX) and 53 genes in Sequenom Mass ARRAY platform (Knight Diagnostics,Portland, OR). Information about mutations in genes other than *FBXW7* discovered in these multiplex panels was also registered.

### Treatment and Evaluation

Patients presenting with *FBXW7* mutations were enrolled, whenever possible, in clinical trials containing inhibitors of the mTOR pathway, particularly protocols testing rapalogs, thought to be primarily anti-mTORC1 agents. Treatment continued until disease progression, withdrawal of consent by the patient, clinical judgment deeming the necessity of removing a patient from a clinical trial, or development of unacceptable toxicity or death.

Clinical assessments were performed as specified in each protocol, typically before the initiation of therapy and then, at a minimum, at the beginning of each new treatment cycle. Treatment response was assessed using computed tomography scans, magnetic resonance imaging and/or positron emission tomography scans at baseline before treatment initiation, and then every 2 cycles (6–8 weeks). All radiographs were read in the Department of Radiology at MD Anderson and reviewed in the Department of Investigational Cancer Therapeutics tumor measurement clinic. Responses were categorized using RECIST on the basis of specific protocol requirements [16], [17], and were reported as best response, defined as the maximum shrinkage of tumor or stabilization of the disease during all the assessments obtained.

### Statistical Analysis

Patient characteristics, including demographics, tumor type, *FBXW7* mutation status and associated genetic abnormalities were summarized using frequency distributions and percentages. Fisher’s exact test was used to assess the association among categorical variables and *FBXW7* mutation status. Time to treatment failure (TTF) was defined as the interval from the start of therapy to treatment discontinuation for any reason, including disease progression, treatment toxicity, patient preference, physician judgment, or death. The Wilcoxon signed rank test assessed TTF differences within patients. All tests were 2-sided, and P<0.05 was considered statistically significant. All statistical analyses were carried out using S+ sofware, ver 8.2 (TIBCO Software Inc, Houston, TX).

## Results

### Patient Characteristics

The tumors from 418 patients with advanced cancer were assessed for *FBXW7* mutations using various multigene panels, and in 17 (4.0%) a mutation in *FBXW7* was identified. Of these 17 patients, 9 (52%) were male and 7 (41%) had colorectal cancer. The median number of prior therapies before initial evaluation in the Phase I Clinic was 2 (0–9). Patient characteristics are summarized in Tables 1 and 2. The following tumor types had more than 10 patients tested and no *FBXW7* mutation detected: adenoid cystic (n = 14), breast cancer (n = 37), gastroesophageal (n = 29), lung (n = 15), kidney (n = 10) and soft tissue sarcoma (n = 61). We tested a mixed of primary and metastatic tumors and of the 17 positive samples 12 (71%) were derived from primary and 5 (29%) from metastatic lesions. The prevalence of *FBXW7* mutation was similar in samples from primary and metastatic tumors.

**Table 1:** Baseline characteristics of *FBXW7* mutation-positive patients and prevalence of associated mutations.

Category	Subcategory	Patients (n = 17)
**Age (years)** Median (range)		60 (16–74)
**Gender (%)**	Male	9 (53)
	Female	8 (47)
**Ethnicity (%)**	White	13 (76)
	African American	2 (12)
	Hispanic	2 (12)
**Tumor type [n/patients tested: (%)]**	Colorectal	7/49 (14.3)
	Head and Neck (squamous)	2/18 (11.1)
	Bladder	1/8 (12.5)
	Cervix	1/10 (10)
	Endometrial	1/7 (14.3)
	Liver	1/13 (7.7)
	Ovarian	1/40 (2.5)
	Mesothelioma	1/4 (25)
	Pancreatic	1/2 (50)
	Teratoma	1/1 (100)
***FBXW7*** ** mutation (%)**	Inactivating	13 (76)
	Unknown function	4 (24)
***KRAS*** ** mutation (%)**	Positive	6 (35)
	Negative	11 (65)
***TP53*** ** mutation (%)**	Positive	10 (59)
	Negative	7 (41)
***PI3KCA*** ** mutation (%)**	Positive	3 (18)
	Negative	14 (82)
***APC*** ** mutation (%)**	Positive	3 (18)
	Negative	14 (82)

**Table 2:** Characteristics of 18 patients with *FBXW7* mutation-positive tumors.

Patient No.	Tumor type	Histology	FBXW7 mutation	Type of aberration	Concomitant mutations	mTOR therapy	Best response	TTF (mos)^a^
1	Bladder	Undifferentiated carcinoma	W244*	Inactivating	*TP53*	Sirolimus, HCQ	SD	2.7
2	Cervix	Squamous	W244*	Inactivating	*CBL, MLH1, PRKDC*	–		
3	Colorectal	Adenocarcinoma	R465C	Inactivating	*KRAS, APC, SMAD4*	–		
4	Colorectal	Adenocarcinoma	R278*	Inactivating	*KRAS, SMAD4*	–		
5	Colorectal	Adenocarcinoma	R505C	Inactivating	*KRAS, TP53*	Everolimus, pazopanib	SD	3
6	Colorectal	Adenocarcinoma	R479Q	Inactivating	*KRAS, TP53*	–		
7	Colorectal	Adenocarcinoma	G499Vfs*25	Unknown	*KRAS*	Sirolimus,HCQ	SD	4.4
8	Colorectal	Adenocarcinoma	R505C	Inactivating	*KRAS, TP53, PIK3CA*	Everolimus, anakinra	PD	1.4
9	Colorectal	Adenocarcinoma	R222*	Inactivating	*TP53, APC, HER-2*	Temsirolimus, bevacizumab, cetuximab	SD	3.6
10	Endometrial	Clear cell	R465C	Inactivating	*TP53, PIK3R1, MAP3K1*	–		
11	Liver	Fibrolamellar HCC	E192A	Unknown	None	Sirolimus, vorinostat	SD	6.8+
12	Head and Neck	Squamous	S282*	Inactivating	*TP53, CDKN2A*	Temsirolimus, bevacizumab, valproic acid	SD	2.2
13	Head and Neck	Squamous	R479Q	Inactivating	*MLL2*	–		
14	Pleura	Mesothelioma	R658*	Unknown	*TP53, NF2*	Sirolimus, lapatinib	PD	1.3
15	Ovarian	Serous	R465H	Inactivating	*TP53, PIK3CA*	Everolimus, Anastrozole	PD	3.3
16	Pancreatic	Neuroendocrine	E113D	Unknown	None	–		
17	Teratoma	Adenocarcinoma	726+1 G>A splice	Inactivating	*TP53, ARID1A*	Temsirolimus, bevacizumab, carboplatin	SD	2.6

Abbreviations- HCQ: hydroxichloroquine; SD: stable disease, PD: progressive disease; PR: partial response; TTF: time to treatment failure, HCC: hepatocellular carcinoma ^a^+ denotes not progressing at the time of the analysis.

### Concomitant Mutations

Genetic abnormalities analyzed using different multiplex gene assays revealed isolated *FBXW7* mutations in 2 of 17 (12%) patients. The most frequent concomitant genetic abnormality was *TP53* mutation in 10 (59%) patients. Other frequently occurring abnormalities were *KRAS*, *PIK3CA* and *APC* mutations (Table 1 and 2).

### Comparison of Clinical and Mutational Characteristics

Because 7 out of 17 (41%) patients with *FBXW7* mutations had colorectal cancer, we compared the clinical, pathological and mutational characteristics between the colorectal cancer population and the non-colorectal cancer patients with mutation (Table 3). In addition, 41 patients with colorectal cancer without *FBXW7* mutation were used as a control group. Of the 10 patients with cancers other than colorectal and *FBXW7* mutations, none (0%) had simultaneous *KRAS* mutations or APC mutations compared to 6/7 (86%) patients with KRAS mutations and 3/7 (43%) with APC mutations in the colorectal cancer group (p = 0.0006 and p = 0.022, respectively Table 3). No statistical differences were observed in sex, ethnicity, age and type of metastasis between these groups.

**Table 3:** Comparison of characteristics of subgroups of *FBXW7*-positive patients and a control group of CRC patients tested negative for a *FBXW7* mutation.

Characteristic	Non-CRC *FBXW7* pos (N = 10) (%)	CRC *FBXW7* pos (N = 7) (%)	CRC *FBXW7* neg (N = 41) (%)
Female	6 (60)	2 (29)	23 (56)
White	8 (80)	5 (71)	27 (66)
Median Age	59 (15–74)	57 (36–67)	52 (23–74)
>3 metastatic sites	1 (10)	1 (14)	6 (15)
Liver metastasis	3 (30)	5 (71)	33 (80)
Lung metastasis	5 (50)	6 (86)	32 (78)
*KRAS* mutation	0 (0)^a^	6 (86)^a^	33 (80)
*TP53* mutation	6 (60)	4 (57)	17 (41)
*APC* mutation	0 (0)^b^	3/5 (60)^b^	16/24 (67)
*PIK3CA* mutation	1 (10)	1 (14)	8 (20)

a p = 0.0006;

b p = 0.022 (Fisher exact test).

CRC: colorectal cancer.

### Treatment of Patients with mTOR Inhibitors

**Figure 1:**
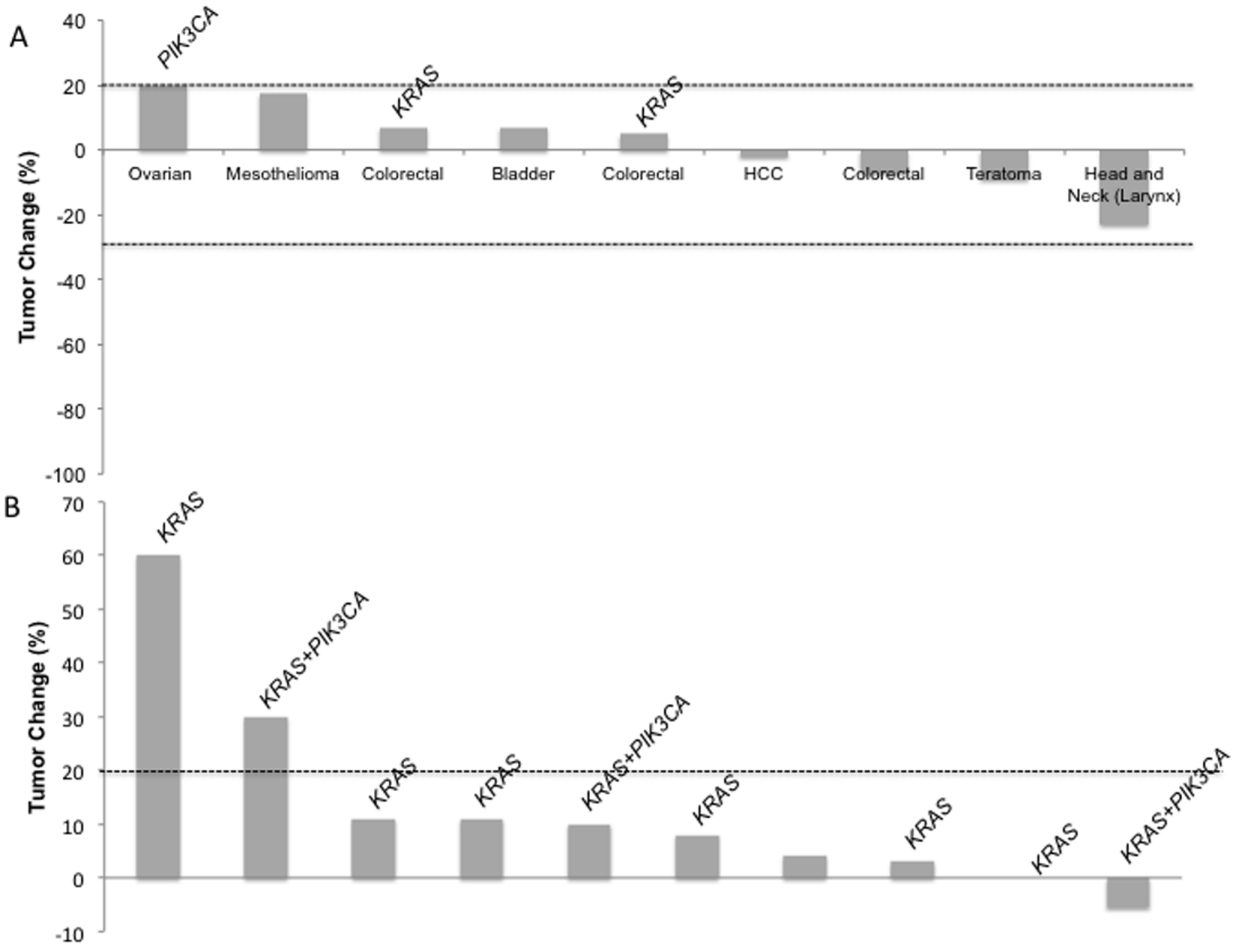
Waterfall plot of patients with measurable disease by RECIST treated with mTOR inhibitors. (A) Responses of patients who were positive for *FBXW7* mutations. (B) Responses of colorectal cancer patients negative for *FBXW7* mutations. Concomitant *KRAS* and *PIK3CA* mutations are indicated.

Of the 17 patients with *FBXW7* mutations, 10 (59%) were enrolled in phase I protocols including mTOR inhibitors combined with other agents (Table 2). All of these patients were evaluable for response. The doses of mTOR inhibitors ranged from 50 to 100% of the maximum tolerated dose or the highest expected dose when the dose escalation was ongoing at the time of analysis. All doses were considered to be active based on prior experience with the drugs. There was no partial or complete response and 7 (70%) had stable disease (SD) as their best response, including 2 (20%) patients with prolonged SD lasting more than 16 weeks (Figure 1).

The median TTF for the cohort of 10 patients with *FBXW7* mutations treated with mTOR inhibitors was 2.8 months (1.3–6.8), including 1 patient still being treated at the time of this analysis. All of these patients had received prior therapies and a trend for an worse TTF on mTOR inhibitor protocols was detected when compared to the treatment immediately before referral to the Phase I Clinic (TTF = 5.7 months [1.3–18] from their prior therapy versus 2.8 months [1.3–6.8] from an mTOR-based treatment, p = 0.055). Of note, the patient with the highest TTF while on an mTOR-based regimen had a tumor that did not harbor *KRAS* mutations. Due to the small sample size, we could not detect a difference in the median TTF on mTOR therapy between patients with a *FBXW7* mutation, with or without concomitant *KRAS* mutations (median TTF = 3.0 and 2.7 months, respectively, p = 0.99).

### Comparison of Outcomes of Colorectal Patients Treated with mTOR Inhibitors

A total of 4 patients with colorectal cancer and a *FBXW7* mutation were treated with an mTOR inhibitor and 3 patients (75%) had SD as their best response. Of the 41 patients with colorectal cancer without a *FBXW7* mutation, 12 (29%) were enrolled on a protocol with an mTOR inhibitor and 4 (33%) had SD (Figure 1). The median TTF of *FBXW7*-positive colorectal cancer patients was 3.3 months (1.4–4.4) on mTOR inhibitors versus 1.8 months (0.6–4.9) for *FBXW7*-negative patients with colorectal cancer (p = 0.27).

## Discussion

*FBXW7* mutations were found in 17 of 418 (4.0%) patients referred to our phase I unit with various advanced tumor types. Although colorectal cancer was not the most prevalent cancer among the patients, it comprised the greatest number of patients testing positive for *FBXW7* mutations. No particular demographic or pathological feature was characteristic of the *FBXW7*-mutated population in our sample, but we found that the mutation rarely occurs in isolation, especially in colorectal cancer. Our series revealed an overall low activity of mTOR inhibitors in patients with *FBXW7* mutations, contrasting with previous pre-clinical data.

Previous studies revealed *FBXW7* mutations in approximately 6% of cancer patients. A substantial variation in the frequency of mutation among tumor types was also described, with the highest mutation rate in cholangiocarcinoma (30%), followed by gastrointestinal tumors (including colorectal cancers), and endometrial and prostate cancers, in the range of 4–15%.[18]–[21].

As of May 2013, the Catalogue of Somatic Mutations in Cancer (COSMIC) database reported an overall frequency of the *FBXW7* mutation in 4% of cancer patients, identical to our data. [22] The highest frequencies were in biliary tumors (22%), endometrial (11%), urinary (8%), and colorectal cancers (7%). Our study demonstrated the highest frequency of the *FBXW7* mutation in colorectal cancer (14.3%). We also detected this mutation in endometrial (1/7 patients) and bladder cancer (1/8 patients), but none of the 3 patients tested with cholangiocarcinoma harbored a *FBXW7* mutation. Of note, our data describe a *FBXW7* mutation in liver cancer, specifically a fibrolamellar variant, and also in rare tumors, such as mesothelioma and teratoma. In COSMIC database tested samples derived from primary tumors, while in our study samples from metastatic tumors were also tested.

*FBXW7* mutations as single molecular abnormalities were rare in our study, which might reflect the difficulty of targeting this mutation with agents blocking single pathways. Indeed, prevalence of concomitant mutations in *TP53* (59%), *KRAS* (35%) and *PIK3CA* (18%) in our patients with *FBXW7* mutations was higher than previously reported with technologies preceding multiplex genomic technologies, which had limited detection capabilities. A prior study described the following prevalence of mutations on the same genes: *TP53*, 37%, *KRAS*, 18% and *PIK3CA* 10%, respectively. [8].

Occurrence of concomitant *KRAS* mutations was more frequently seen in colorectal cancer patients with *FBXW7* mutations in comparison to non-colorectal group. The analysis of a control group of colorectal patients who tested negative for a *FBXW7* mutation showed that this association was, in fact, due to the high prevalence of *KRAS* mutations in our colorectal cancer population and, therefore, we cannot conclude about any possible association between these mutations.

We described a similar previously reported [18] pattern of the *FBXW7* mutation available in the COSMIC database. The vast majority of the mutations detected in our study were single nucleotide changes, predominantly missense substitutions. Of these, the most common mutations were found in two mutational hotspots in the Arg^465^ and Arg^479^ codons. Interestingly, our series of patients with more advanced and refractory solid tumors had a higher frequency of nonsense substitutions than previously described. [18] Since the functional consequences of both types of substitutions are not well compared in the literature it is hard to speculate about the possible implications of this finding.

Considering that most of these mutations are within the WD40 domain responsible for the recognition of substrates by *FBXW7* or result in a stop of the translation process prior to inclusion in this domain, *FBXW7* mutations are expected to inactivate the translated protein. [11] Most *FBXW7* mutations are heterozygous, but because the protein dimerizes, how they affect protein functionality varies. [23] Mutations that result in retention of the dimerization domain may have a dominant negative effect that is more deleterious than mutations resulting in allele deletions or premature stop codons. [11] Hence, similar to other druggable oncogenic aberration, it is anticipated that the functional consequences of *FBXW7* mutations upon substrates are quite variable, and responses to targeted therapies might also be heterogeneous. [24], [25].

When we evaluated the response to mTOR inhibitors in a subset of patients with tumors harboring *FBXW7* mutations, variable responses were demonstrated. Previous studies showed that tumor cell lines with *FBXW7* gene mutations are sensitive to rapamycin [14], suggesting a potential rationale for treating such tumors with mTOR inhibitors. Overall, however, we found only limited activity in phase I trials using mTOR inhibitors in this population. The TTF for the overall population was 2.8 months, which was worse than the TTF on the therapies used immediately prior to enrollment on phase I protocols with mTOR inhibitors. The absence of tumor responses with mTOR inhibitors also indicates a lack of activity of mTOR inhibitors for *FBXW7* positive patients. It is important to note that our data is limited to mTORC1 inhibitors, which have some concerns related to re-activation of mTOR pathway by reversal of a feed-back loop. [26].

Of interest, one patient with a refractory fibrolamellar hepatocellular carcinoma demonstrated prolonged SD for 6.8 months on sirolimus based combination and is still on treatment at the time of this report. This patient was the only one among those treated with mTOR inhibitors who had *FBXW7* mutation without other simultaneous molecular abnormalities. It is plausible that similar to other malignancies simultaneous molecular aberrations can lead to activation of other molecular pathways, which can lead to therapeutic resistance. [24], [27] Specifically for mTOR inhibitors, it has been shown that *KRAS* mutation is a mechanism of resistance even in the presence of sensitizing mutations, such as the *PIK3CA* mutations. [24], [28], [29] Therefore, absence of simultaneous mutations along with better understanding of functional consequences of specific mutation types can be crucial for patient selection for mTOR inhibitors.

One study suggested that rapalogs can delay *FBXW7*-induced tumorigenesis [30], but it is not clear whether these drugs produce a cytoreductive effect. Obtaining disease stabilization as best response in our study points to a direction where mTOR inhibitors are unable to produce tumoral reductions of *FBXW7* positive tumors. In addition, the absence of responses in our population combined with a short period of disease control suggests again that pathways other than PI3K/AKT/mTOR confer resistance to mTOR-based therapies. In contrast to preclinical models were *FBXW7* are studied as an isolated event, we showed that the predictive information generated with these models do not translate necessarily to the *in vivo* context. There are many possible reasons (including association of concomitant mutations, stromal effects among others) that preclude the validity of the preclinical data for treatment-based decisions. These models are essentially hypothesis generating and should encourage the development of clinical protocols to test the potential findings that they generate.

The limitations of this study are its retrospective nature, small sample size, and inclusion of protocol treatments that were not exclusively mTOR inhibitors. Additionally, a possible selection bias and absence of randomization to study drugs characterizes this study as exploratory in nature. However, to our knowledge, this is the first series reporting the clinical characteristics and treatment outcomes of an *FBXW7*-mutated cancer patient population. Additional series are needed to better understand therapeutic alternatives that can be used to target this mutation, by exploring use of alternatives to mTOR-1 inhibition and correlating *FBXW7* mutations with mTOR pathway activation *in vivo*. That is a strong possibility given that several oncogenes are regulated by *FBXW7*, including MYC, Notch, JUN and cyclin E. Thus, targeting different pathways in addition to the mTOR pathway might be necessary to effectively kill cancer cells in tumors driven by *FBXW7* alterations. [11].

In conclusion, we demonstrated that *FBXW7* mutations are found in several types of solid tumors. In addition, the diverse types of *FBXW7* mutations are likely related to various functional effects that the *FBXW7* mutation has on protein functionality. Also, the frequent concomitance of other oncogene mutations provides challenges to targeting tumors harboring *FBXW7* abnormalities. Most patients with *FBXW7* mutations had limited benefit from mTOR based therapies; however, studying mTOR inhibitors in cancers lacking simultaneous molecular abnormalities as well as describing functional consequences of specific *FBXW7* mutation subtypes warrants further investigation.
